# Personal

**Published:** 1896-11

**Authors:** 


### Personal.

Dr. P. M. Wise, formerly of Clarence, Erie County, N. Y., has been appointed state commissioner in lunacy vice Dr. Carlos F. McDonald resigned.

Dr. Wise was educated in the common schools, at Clarence Academy and at the Parker Classical Institute. He was graduated in medicine at the Buffalo University Medical College in 1872, and entered the St. Louis City Hospital as interne. During the smallpox epidemic of the following winter he was appointed resident physician of the smallpox hospital. In 1873, he was appointed an assistant physician at the Willard Asylum for the Insane, and so remained until 1884, when, upon the resignation of Dr. Chapin, he was unanimously elected to fill the vacancy as medical superintendent. He occupied this position for six years, and until he resigned to accept a similar position at the St. Lawrence Hospital, which he has occupied until the present time.

For the last five years he has been professor of mental diseases in the University of Vermont. He is the author of a Text-book for training-schools for nurses, in two volumes.

---

Dr. John B. Hamilton, of Chicago, editor of the *Journal of the American Medical Association*, has resigned his commission as surgeon in the U. S. Marine Hospital Service. Dr. Hamilton is to be congratulated on relieving himself of an incubus that could not be otherwise than disagreeable. He will now devote his entire attention to his large surgical practice and to editorial work.

---

Dr. John T. Pitkin announces that he is prepared to perform all manner of X-ray work, flouroscopic or skiographic, of any portion of the human bony framework or foreign bodies in its addenda. He is located at 388 Ellicott Square and invites inspection of his apparatus and work.

---

Dr. Jacob Goldberg, of Buffalo, has removed his office and residence from 145 Cedar street to 329 Eagle street. Dr. Goldberg has prepared his office for special work in diseases of the eye and ear. Hours: 9–12 A. M., 3–5 and 7 P. M. Telephone, Howard 222—A.

---

Dr. Frank P. Vandenbergh has been appointed commissioner of the State of New York to the Tennessee Centennial Exposition at Nashville, in 1897.

---

Dr. William G. Bissell has removed from the corner of Porter and Normal avenues to the Buckingham, corner Allen and Mariner streets.

